# Prediction of hyperuricemia in people taking low-dose aspirin using a machine learning algorithm: a cross-sectional study of the National Health and Nutrition Examination Survey

**DOI:** 10.3389/fphar.2023.1276149

**Published:** 2024-01-19

**Authors:** Bin Zhu, Li Yang, Mingfen Wu, Qiao Wu, Kejia Liu, Yansheng Li, Wei Guo, Zhigang Zhao

**Affiliations:** ^1^ Department of Pharmacy, Beijing Tiantan Hospital, Capital Medical University, Beijing, China; ^2^ Emergency Department, Beijing Tiantan Hospital, Capital Medical University, Beijing, China; ^3^ DHC Mediway Technology Co., Ltd., Beijing, China; ^4^ Beijing Hospital of Traditional Chinese Medicine, Capital Medical University, Beijing, China

**Keywords:** Hyperuricemia, National Health and Nutrition Examination Survey, Aspirin, machine learning, eXtreme Gradient Boosting

## Abstract

**Background:** Hyperuricemia is a serious health problem related to not only gout but also cardiovascular diseases (CVDs). Low-dose aspirin was reported to inhibit uric acid excretion, which leads to hyperuricemia. To decrease hyperuricemia-related CVD, this study aimed to identify the risk of hyperuricemia in people taking aspirin.

**Method:** The original data of this cross-sectional study were obtained from the National Health and Nutrition Examination Survey between 2011 and 2018. Participants who filled in the “Preventive Aspirin Use” questionnaire with a positive answer were included in the analysis. Six machine learning algorithms were screened, and eXtreme Gradient Boosting (XGBoost) was employed to establish a model to predict the risk of hyperuricemia.

**Results:** A total of 805 participants were enrolled in the final analysis, of which 190 participants had hyperuricemia. The participants were divided into a training set and testing set at a ratio of 8:2. The area under the curve for the training set was 0.864 and for the testing set was 0.811. The SHapley Additive exPlanations (SHAP) method was used to evaluate the performances of the modeling. Based on the SHAP results, the feature ranking interpretation showed that the estimated glomerular filtration rate, body mass index, and waist circumference were the three most important features for hyperuricemia in individuals taking aspirin. In addition, triglyceride, hypertension, total cholesterol, high-density lipoprotein, low-density lipoprotein, age, race, and smoking were also correlated with the development of hyperuricemia.

**Conclusion:** A predictive model established by XGBoost algorithms can potentially help clinicians make an early detection of hyperuricemia risk in people taking low-dose aspirin.

## Introduction

Hyperuricemia is generally caused by an increase in the concentration of serum uric acid (SUA), which has been considered the key factor of gout (Dalbeth et al., 2021). It is estimated that approximately 38 million adults or 16.9% of the population in the United States are affected by hyperuricemia (Leung et al., 2022). Concurrently, the estimated hyperuricemia prevalence among Chinese adults was 14.0% (Zhang et al., 2021). As an important worldwide public health issue, hyperuricemia is associated with a state of oxidative stress that promotes a pro-inflammatory state and produces endothelial dysfunction, which can contribute to a variety of comorbidities including artery atherosclerosis, hypertension, chronic kidney disease, and metabolic syndrome (Zhang et al., 2019; Agnoletti et al., 2021; Padda et al., 2021). Accumulating evidence reveals that hyperuricemia is associated with a higher risk of 10-year cardiovascular diseases (CVDs). Moreover, increased SUA levels have been independently and significantly linked to the risk of mortality caused by cardiovascular and cerebrovascular diseases (Fang and Alderman, 2000; Chen et al., 2022; Wei et al., 2022).

Hyperuricemia can be attributed to the increased generation of uric acid or decreased uric acid excretion. Many factors such as renal problems and dietary factors (purine-rich foods and drinking) contribute to these two aspects (Petreski et al., 2020). Additionally, it is noteworthy that multiple medications also increase SUA levels and play an important role in the pathogenesis of hyperuricemia (Ben et al., 2017). Aspirin, which is widely used in the prevention of atherosclerotic cardiovascular disease, has been reported to reduce uric acid excretion and may induce hyperuricemia (Leung et al., 2022). As a non-steroidal anti-inflammatory drug, aspirin showed a biphasic effect on SUA levels, wherein low doses can increase the SUA level, while high doses decrease the SUA level. This paradoxical effect has led to the cautionary use of aspirin in patients with a history of gout or renal problems (Segal et al., 2006; Zhang et al., 2014).

Although studies revealed no significant impact of aspirin on hyperuricemia, hyperuricemia was reported to cause aspirin resistance, which may lead to failure in the primary prevention of heart disease (Wong et al., 2004; Li et al., 2021). It has been documented that approximately 20%–30% of patients are resistant to their aspirin therapy, which increases the risk of adverse cardiovascular events by almost three-fold in various patient populations (Khan et al., 2022). As hyperuricemia is often asymptomatic, and indications for initiating treatment are not definitive (Stone et al., 2019), it is crucial to identify individuals at high risk of hyperuricemia, especially those who take aspirin for a long term.

Currently, machine learning (ML) algorithms are gaining popularity in addressing complex problems of healthcare decision making. In this study, by using the data from the National Health and Nutrition Examination Survey (NHANES), we aim to make use of a machine learning method to develop a prediction model to identify hyperuricemia risk in individuals taking low-dose aspirin.

## Materials and methods

### Study population

Data for the analysis were collected from the NHANES (https://www.cdc.gov/nchs/nhanes/index.htm) around 2011–2018 (covered four periods: 2011–2012, 2013–2014, 2015–2016, and 2017–2018). As a public database, the NHANES is a nationally representative survey that assesses the health and nutrition status of the US non-institutionalized civilian population. Participants over 40 years of age who answered the questionnaire “Preventive Aspirin Use” with a positive answer were enrolled in our analysis. All individual privacy is kept strictly confidential, and the NCHS Research Ethics Review Board approved all NHANES protocols of the survey (https://www.cdc.gov/nchs/nhanes/irba98.htm).

Sociodemographic information and relative laboratory parameters were extracted from the database year by year. The hyperuricemia criteria were defined as SUA ≥6.0 mg/dL in females and ≥7.0 mg/dL in males (Yu et al., 2020). The estimated glomerular filtration rate (eGFR) was calculated using the CKD-EPI creatinine equation (2021). Other illness statuses such as hypertension and type 2 diabetes of the participants were determined according to the guidelines, combined with the drug they were taking and the self-reported questionnaire (Whelton et al., 2018; Committee, 2022). Smoking, drinking, and physical activity status was obtained from the corresponding questionnaire data and defined according to the relative criteria published in our earlier study (Zhu et al., 2023).

### Machine learning algorithm

All participants were randomly divided into training and testing sets. Six machine learning algorithms, namely, logistic regression, random forest, adaptive boosting, light gradient-boosting machine, category boosting, and eXtreme gradient boosting (XGBoost), were utilized to identify the optimal performing model. The receiver-operating characteristic (ROC) curve was used to validate the modeling efficiency, and the area under the curve (AUC) was calculated to evaluate the performance of the ML algorithms between the training and testing sets. To further improve the predictive value of the model, we also used the recursive feature elimination (RFE) method to screen the most important variables that can influence the modeling efficacy (this method involves two main components: RFE, which rates the importance of features through elimination, and cross-validation, which determines the optimal number of features through cross-validation after feature ranking). Additionally, the SHapley Additive exPlanations (SHAP) method, which calculates the marginal contributions of variables, was employed to interpret and rank the importance of each selected variable.

### Statistical analysis

The data are presented as the means with standard deviations, while categorical variables were presented as percentages. Fisher’s exact test or an x^2^ test was conducted for binary variables, and Student’s t-test or a Mann–Whitney *U* test was used for the continuous variables. The XGBoost algorithm was developed and validated using Python software (version 3.8). Statistical significance was set at *p* ≤ 0.05.

## Results

### Basic characteristics

There were 15,063 participants who answered the questionnaire “Preventive Aspirin Use” from 2011 to 2018. After further screening, 805 participants, who were taking aspirin for treating or preventing diseases, were finally enrolled for additional analysis. Of the 805 people, 190 participants were found to have hyperuricemia. The average age of participants without hyperuricemia was 65.0 ± 10.07 years and 66.0 ± 10.68 years for those with hyperuricemia. The eGFR level in the two groups was nearly within the normal range, which was 88.27 ± 16.73 or 70.15 ± 20.68, respectively (*p* < 0.01). In addition, the values of waist circumference, body mass index (BMI), triglyceride (TG), and hypertension rate were much higher in hyperuricemia participants than in those without hyperuricemia (*p* < 0.01). The detailed basic demographic characteristics are shown in Table 1.

**TABLE 1:** Basic characteristics of the enrolled participants who were taking aspirin for treatment or prevention.

Variable	All (n = 805)	Negative (n = 615)	Positive (n = 190)	*p*-value
Age	65.0 ± 10.22	65.0 ± 10.07	66.0 ± 10.68	0.088
Gender (male) (%)	437 (54.29)	335 (54.47)	102 (53.68)	0.915
BMI	28.7 ± 6.27	28.0 ± 5.53	31.65 ± 7.45	<0.01
Waist circumference	103.0 ± 14.48	101.5 ± 13.44	108.60 ± 15.74	<0.01
TC	177.0 ± 44.37	176.0 ± 43.03	178.5 ± 48.22	0.075
LDL	98.0 ± 37.90	97.0 ± 35.78	99.0 ± 43.73	0.038
HDL	51.0 ± 16.91	51.0 ± 17.49	48.5 ± 14.32	<0.01
TG	107.0 ± 65.80	103.0 ± 64.08	128.5 ± 68.68	<0.01
eGFR	84.85 ± 18.55	88.27 ± 16.73	70.15 ± 20.68	<0.01
Diabetes (%)	331 (41.12)	242 (39.35)	89 (46.84)	0.08
Coronary heart disease (%)	124 (15.4)	92 (14.96)	32 (16.84)	0.608
Stroke (%)	75 (9.32)	57 (9.27)	18 (9.47)	0.954
Hypertension (%)	679 (84.35)	502 (81.63)	177 (93.16)	<0.01
Physical_activity (%)	333 (41.37)	262 (42.6)	71 (37.37)	0.232
Ethnicity				
Mexican American (%)	83 (10.31)	63 (10.24)	20 (10.53)	0.08
Other Hispanic (%)	80 (9.94)	62 (10.08)	18 (9.47)	
Non-Hispanic White (%)	348 (43.23)	276 (44.88)	72 (37.89)	
Non-Hispanic Black (%)	178 (22.11)	122 (19.84)	56 (29.47)	
Other race (%)	116 (14.41)	92 (14.96)	24 (12.63)	
Smoking				
Never smoker (%)	397 (49.32)	300 (48.78)	97 (51.05)	0.029
Former smoker (%)	279 (34.66)	205 (33.33)	74 (38.95)	
Current smoker (%)	129 (16.02)	110 (17.89)	19 (10.0)	
Drinking (%)	111 (13.79)	87 (14.15)	24 (12.63)	0.683

### Model performance

**FIGURE 1:**
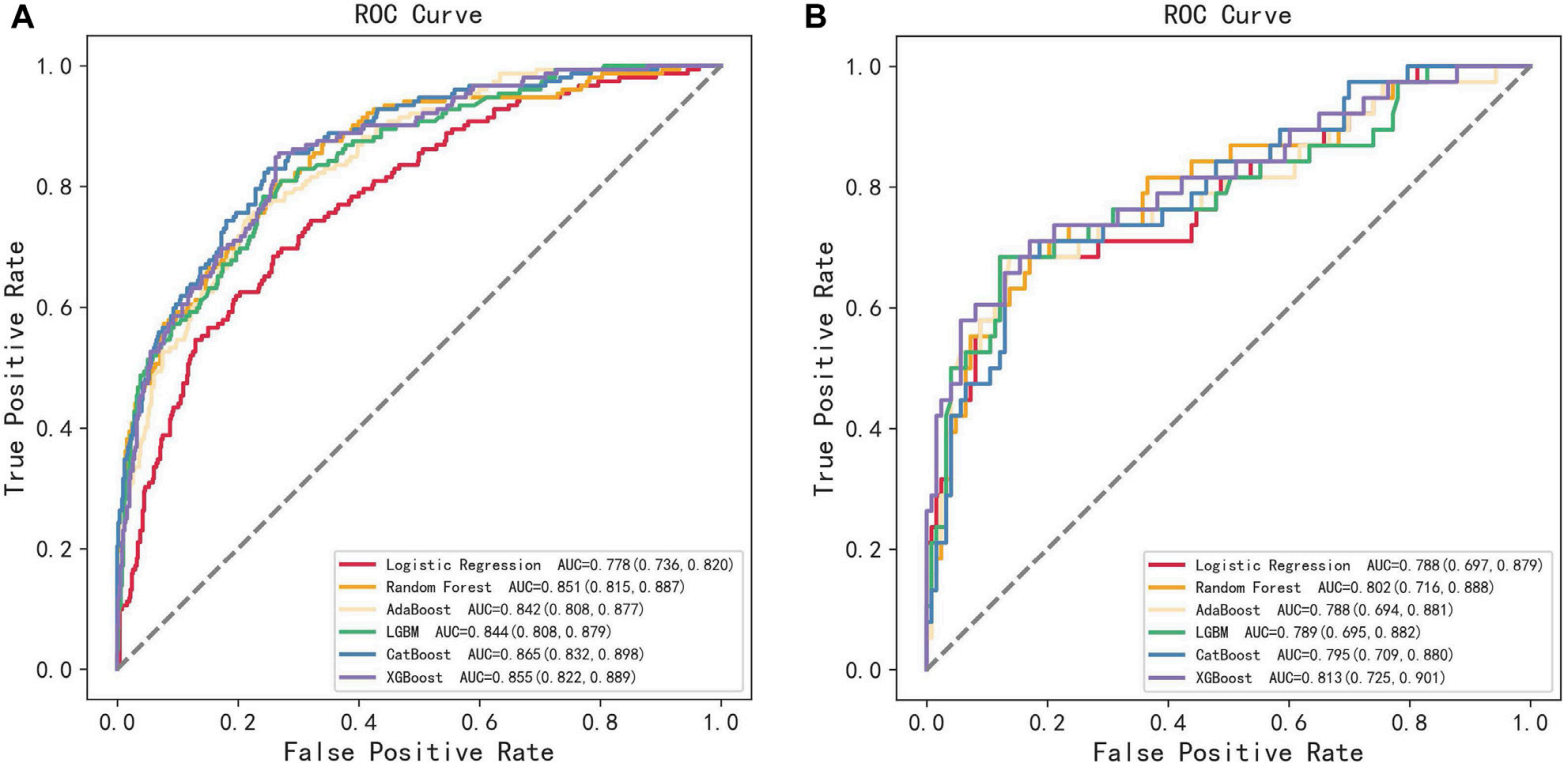
ROC curves of the training set and the testing set obtained by the six machine learning algorithms. **(A)** Training set and **(B)** testing set.

**FIGURE 2:**
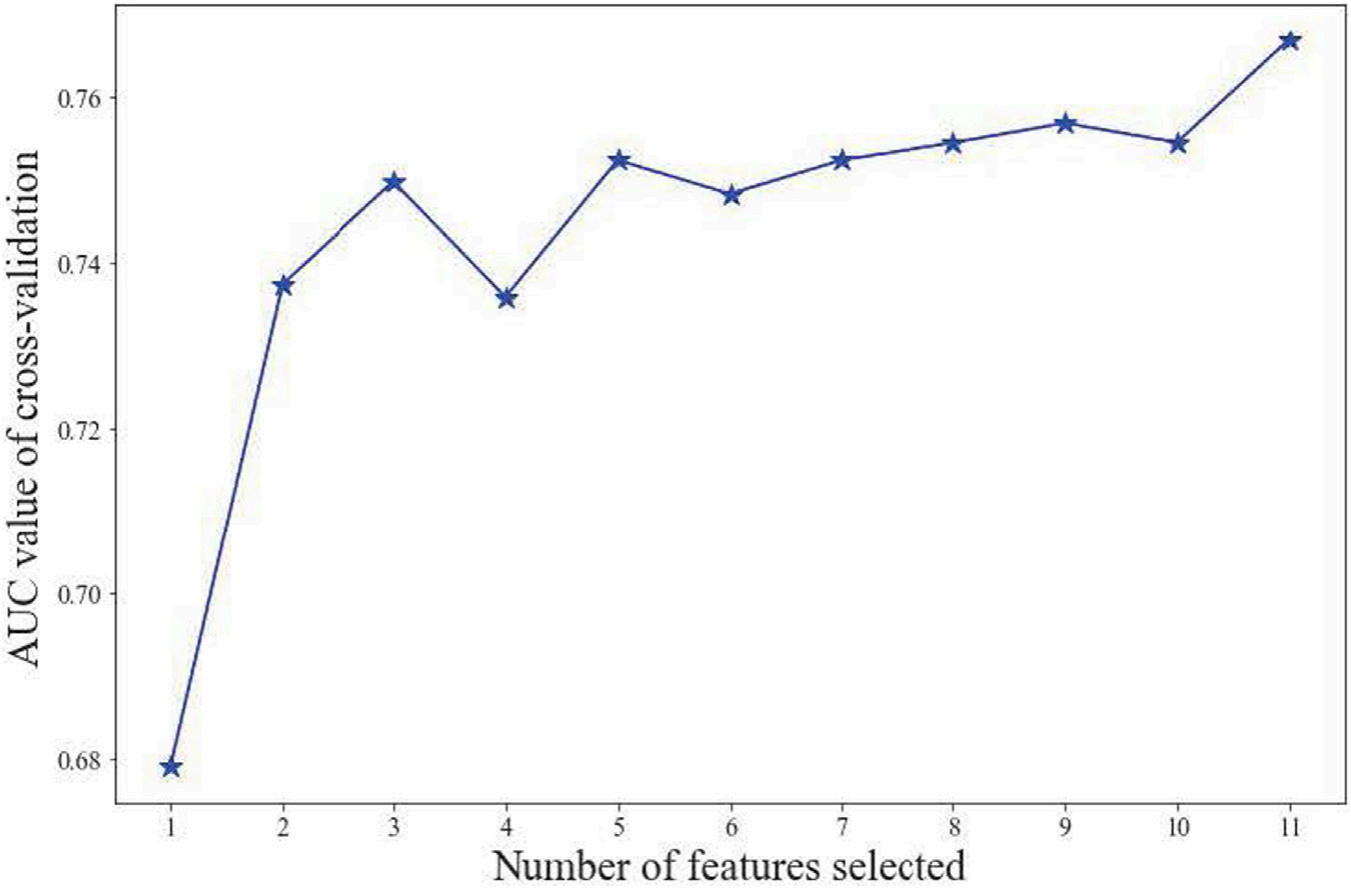
Screening of the most important variables by using the RFE method.

**FIGURE 3:**
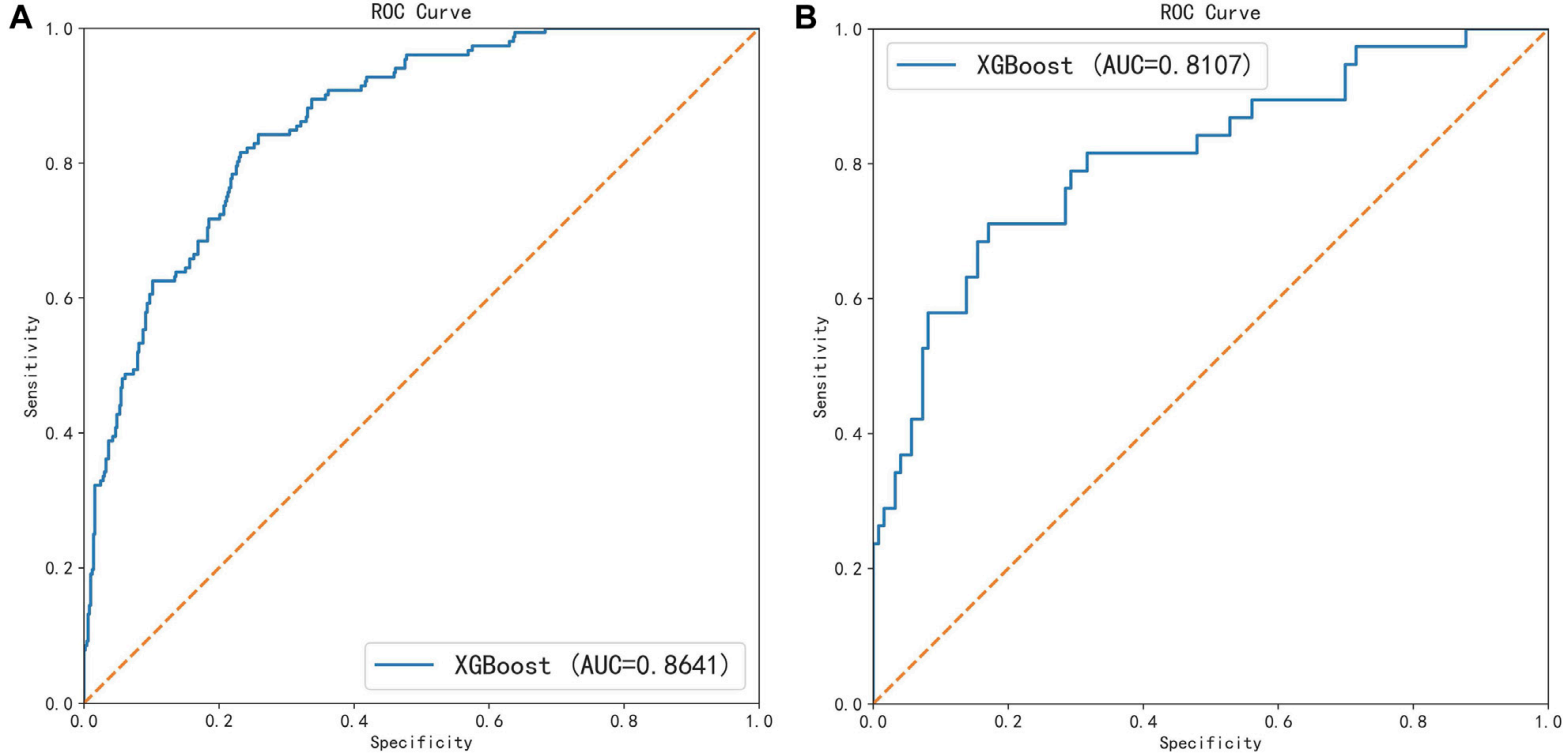
ROC curves of the training set and the testing set under the XGBoost algorithm. **(A)** Training set and **(B)** testing set.

All participants were randomly divided into training and testing sets at a ratio of 8:2. After screening for six machine learning algorithms, the XGBoost algorithm was chosen for the final modeling (Figure 1). A total of 11 variables, namely, age, race, BMI, waist circumference, TG, low-density lipoprotein (LDL), high-density lipoprotein (HDL), total cholesterol (TC), eGFR, hypertension, and smoking, were chosen for modeling after being screened by the RFE algorithm (Figure 2). The modeling results showed that the AUC for the training set was 0.864, and after further evaluation by the testing set, it was 0.811 (Figure 3). In addition, the sensitivity, specificity, positive predictive value (PPV), negative predictive value (NPV), Matthew’s correlation coefficient (MCC), and Kappa in XGBoost modeling for the training and testing dataset are shown in Table 2.

**TABLE 2:** Established prediction model with 11 variables by using the XGBoost algorithm.

	Sensitivity	Specificity	PPV	NPV	MCC	Kappa
Training set	0.684	0.821	0.542	0.894	0.469	0.463
Testing set	0.632	0.846	0.558	0.881	0.458	0.456

### Interpretation and evaluation of the machine learning model

**FIGURE 4:**
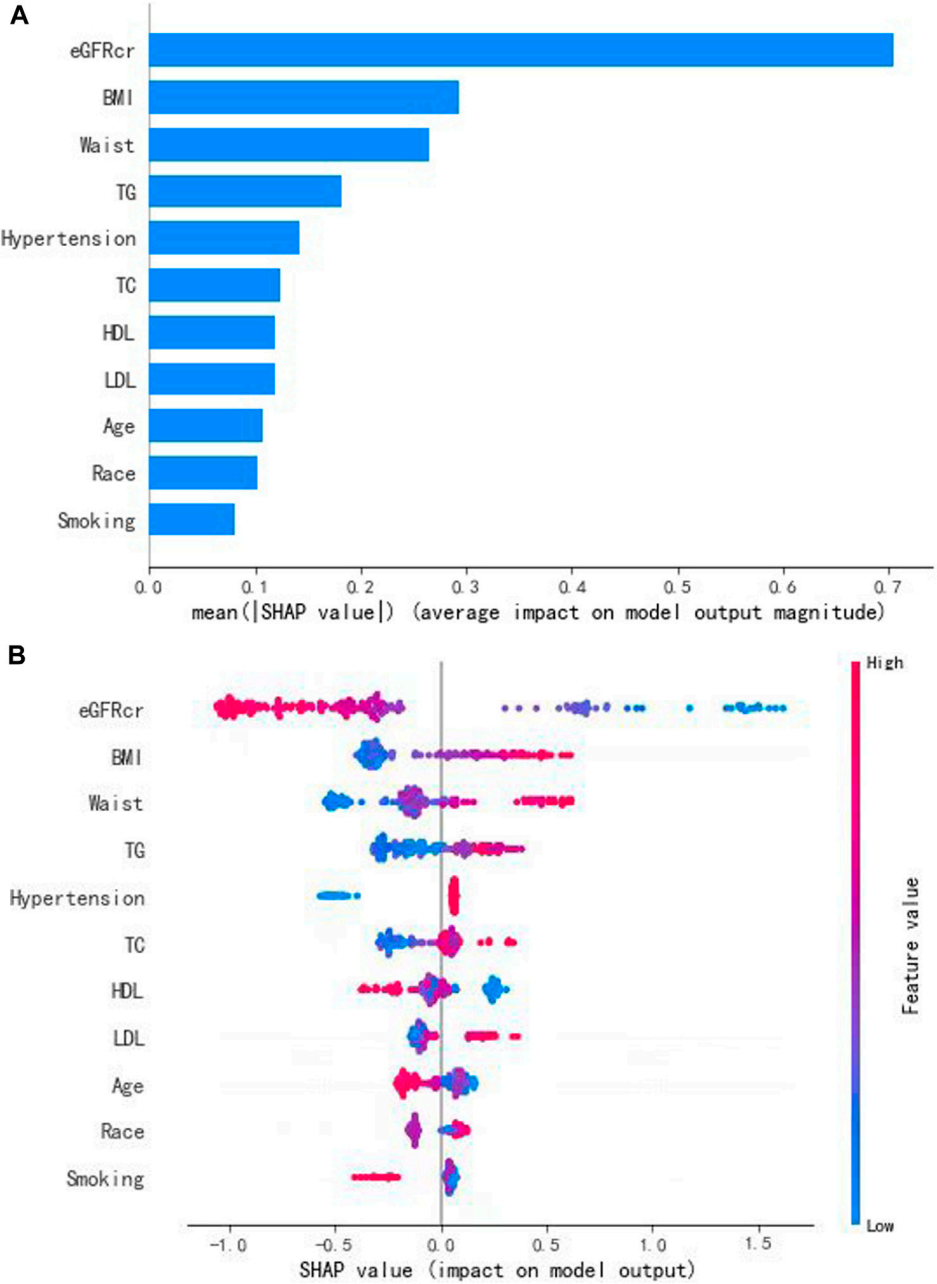
Interpretation and evaluation of the model, which was modeling by the XGBoost algorithm. **(A)** Ranking of feature importance indicated by SHAP. **(B)** SHAP method used to evaluate the performances of the modeling.

To interpret the prediction achieved by the XGBoost model, the SHAP method was used to evaluate the performances of the modeling. Based on the SHAP results, the feature ranking interpretation showed that the eGFR, BMI, and waist circumference were the three most important features for hyperuricemia in individuals taking aspirin. In addition, TG, hypertension, TC, HDL, LDL, age, race, and smoking were also correlated with the development of hyperuricemia (Figure 4).

## Discussion

Aspirin, which has been widely recommended by European and American guidelines, is the cornerstone of antithrombotic management among patients with potential risk factors for cardiovascular and cerebrovascular diseases (Davidson et al., 2022; Guirguis-Blake et al., 2022). However, some studies had reported that low-dose aspirin may also increase SUA levels and result in hyperuricemia, which limited its application. Thus, evaluating the risk factors which may cause hyperuricemia in individuals taking low-dose aspirin for a long time is of great importance. By using the XGBoost algorithm, we established a model with 11 variables to predict the hyperuricemia risk. This study demonstrated that machine learning models can predict hyperuricemia in people taking low-dose aspirin.

Owing to the existence of unexpected potential heterogeneous variables and the inherent noise of data encountered in clinical care, several strategies have been investigated to inform decision making. ML algorithms that leverage statistical methods to learn key patterns from clinical data are gaining extensive popularity (Handelman et al., 2018). As a crucial branch of artificial intelligence, ML algorithms deal with medicine’s multi-modal data (such as clinical, genetic, and many other laboratory outputs) to obtain a greater understanding of human health and disease (Ng et al., 2023). Benefiting from this method, clinicians can identify patients with an undiagnosed disease or those at risk of future disease much earlier and with better predictive accuracy than before (Oikonomou et al., 2022). Previous studies have externally validated the possibility of ML algorithms in addressing complex problems of healthcare decision making under a clinical scenario. Guan et al. used a hybrid machine learning framework to improve the prediction of all-cause rehospitalization among elderly patients in Hong Kong (Guan et al., 2023). Mahajan et al. established an ML model to identify patients at high risk of adverse outcomes prior to surgery, which made perioperative care much more individualized and improved patient outcomes (Mahajan et al., 2023). Furthermore, Lee et al. found that the machine learning model has the potential to empower trained operators to estimate gestational age with higher accuracy in a cohort of 3,842 participants (Lee et al., 2023).

Hyperuricemia is one of the serious health problems not only for individuals with gout but also for those with cardiovascular diseases. Chen et al. reported that hyperuricemia was related to a higher risk of 10-year CVD (Chen et al., 2022). Stone et al. also reported that increased SUA concentration was associated with significantly increased odds of heart failure (Stone et al., 2019). However, except for some traditional risk factors such as a sedentary lifestyle, increased intake of high-protein and high-purine foods, and drinking, drug-induced hyperuricemia also presents an emergent and increasingly prevalent problem in clinical practice. Aspirin can bind to cyclooxygenase and block the synthesis of thromboxane A2, thus inhibiting platelet aggregation. Some studies had reported that low-dose aspirin was associated with hyperuricemia, but others did not (Zhang et al., 2014; Leung et al., 2022). This inconsistency may be attributed to the differences in primary and combined diseases, age, types of medications, and other confounding factors, which lead to patients taking aspirin presenting highly variable effects. The current study pursued the investigation and refinement of ML algorithms for the accurate prediction of hyperuricemia in individuals taking low-dose aspirin.

Although we established a predictive model for hyperuricemia in individuals taking low-dose aspirin, due to the “black box” characteristic, the predictive value of ML should be treated with caution, and it cannot substitute the clinical judgment of a medical professional (Finlayson et al., 2021). In addition, as the sensitivity in our model was lower than specificity, more data, in addition to continuously optimizing and upgrading the model, are needed.

In our study, certain limitations should also be noted. First, this was a cross-sectional study with a small sample size, which may influence the model’s predictive efficiency. Although the total number of participants in the NHANES was large, not enough people were satisfied with our criteria, and further validation in larger sample size is required. Second, most of the variables we used for modeling were from the laboratory index; nevertheless, diet/dietary supplement exposure, living style, or drugs besides aspirin were also important confounding factors for hyperuricemia development. Third, we do not know the duration of people taking low-dose aspirin. This limited the application of our predictive model.

## Conclusion

We leveraged an ML model trained on NHANES data to establish a hyperuricemia model for individuals taking aspirin. The results showed that the XGBoost model can potentially help clinicians make an early detection of hyperuricemia risk in general clinical practice. Future studies are warranted to assess whether this prediction model would decrease hyperuricemia occurrence in people taking low-dose aspirin.

## Data Availability

The original contributions presented in the study are included in the article/Supplementary Material; further inquiries can be directed to the corresponding authors.
